# Supplementary material for: Reprogramming mechanism dissection and trophoblast replacement application in monkey somatic cell nuclear transfer
Source: Nat Commun. 2024 Jan 16;15:5. doi: 10.1038/s41467-023-43985-7 (PMC10791636; doi:10.1038/s41467-023-43985-7)
Supplement: Supplementary file 1 — Supplementary Information [file 41467_2023_43985_MOESM1_ESM.pdf]

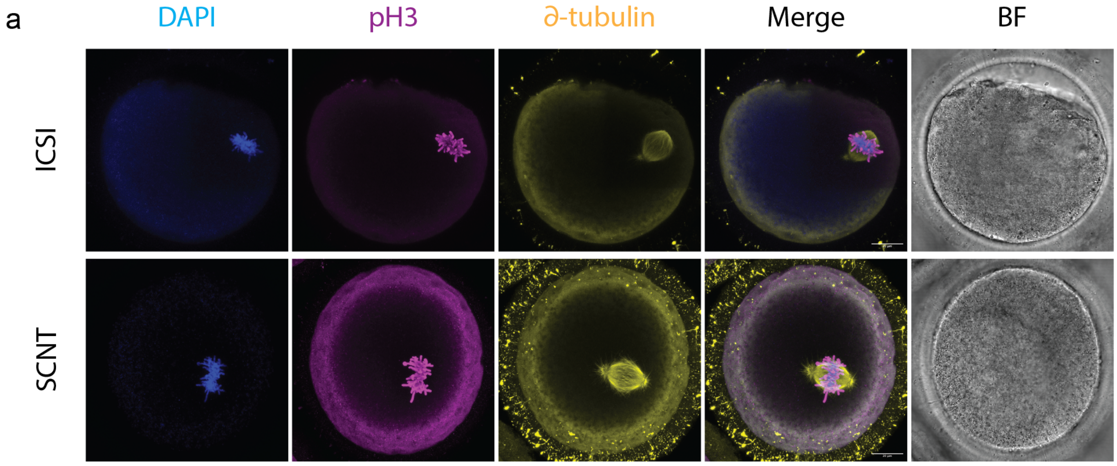

Supplementary Figure 1. **Examination of the M-phase chromosome organization in monkey SCNT embryos.**

a Immunostaining revealing the chromosome organization of SCNT embryos during their first M-phase. BF, bright field images. ICSI group, n=13 independent embryos. SCNT group, n=19 independent embryos.

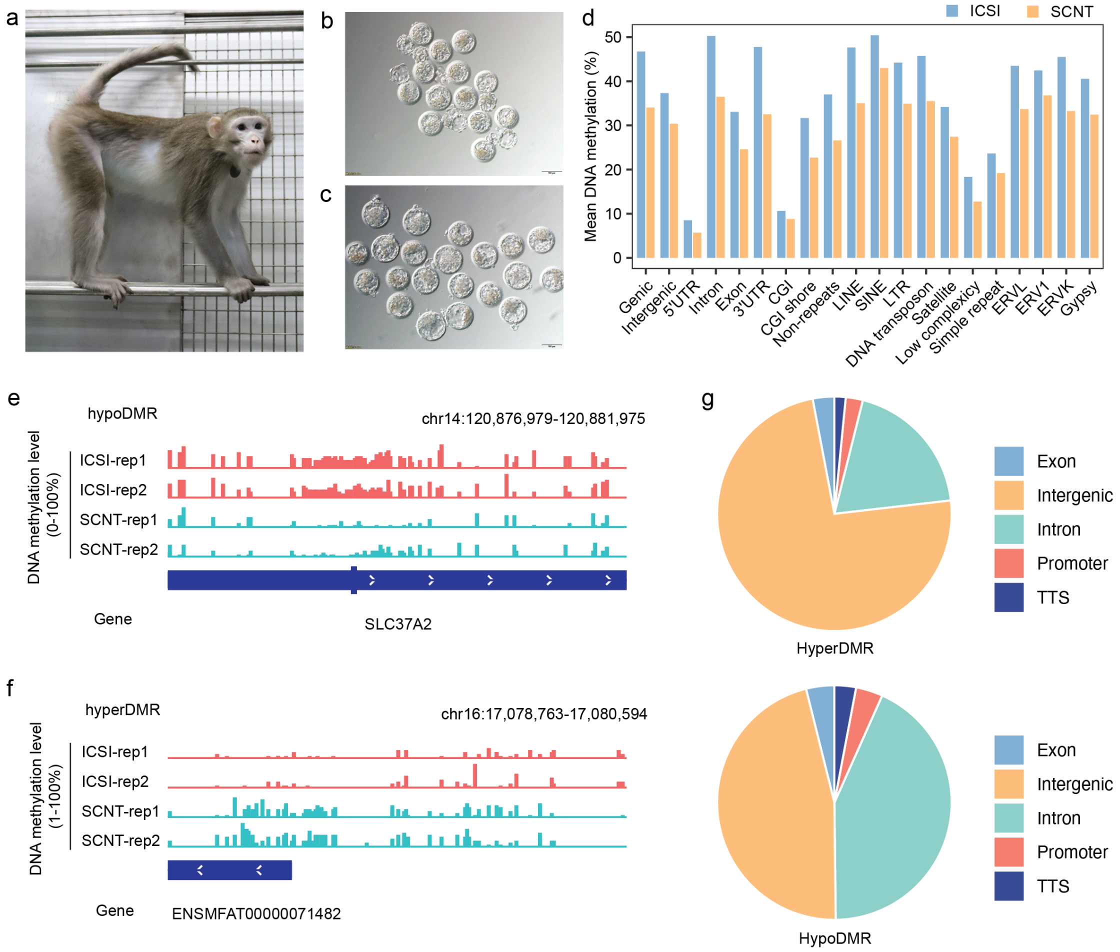

Supplementary Figure 2. **DMRs in hybrid monkey ICSI and SCNT embryos.**

a Hybrid monkey utilized to derive fibroblasts for generating hybrid SCNT blastocysts.

b Hybrid SCNT blastocysts produced using the hybrid fibroblasts.

c Hybrid ICSI blastocysts generated through the usage of sperm from rhesus monkey and oocytes from cynomolgus monkeys.

d Bar plot comparing the overall DNA methylation levels of ICSI and SCNT blastocysts across various genomic elements.

e Genome browser view displaying representative hypoDMRs.

f Genome browser view displaying representative hyperDMRs.

g Pie chart depicting the distribution of hyperDMRs and hypoDMRs in genome. TTS, transcription termination site (default defined from -100 bp to +1 kb).

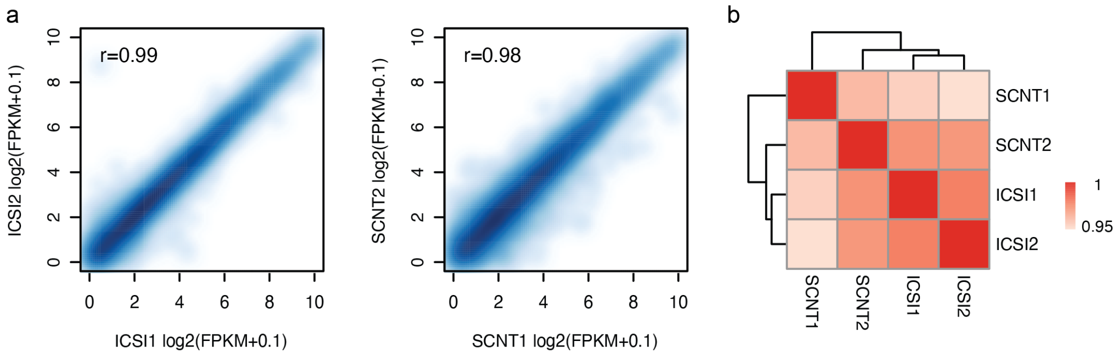

Supplementary Figure 3. **Evaluation of the RNA-seq dataset of ICSI and SCNT blastocysts.**

a Scatter plots comparing transcriptomes of biological replicates of ICSI (left) and SCNT (right) blastocysts. *r* value in the figure indicates the Pearson correlation coefficient between two replicates within each group.

b Heatmap displaying the correlations between the transcriptomes of ICSI and SCNT blastocysts.

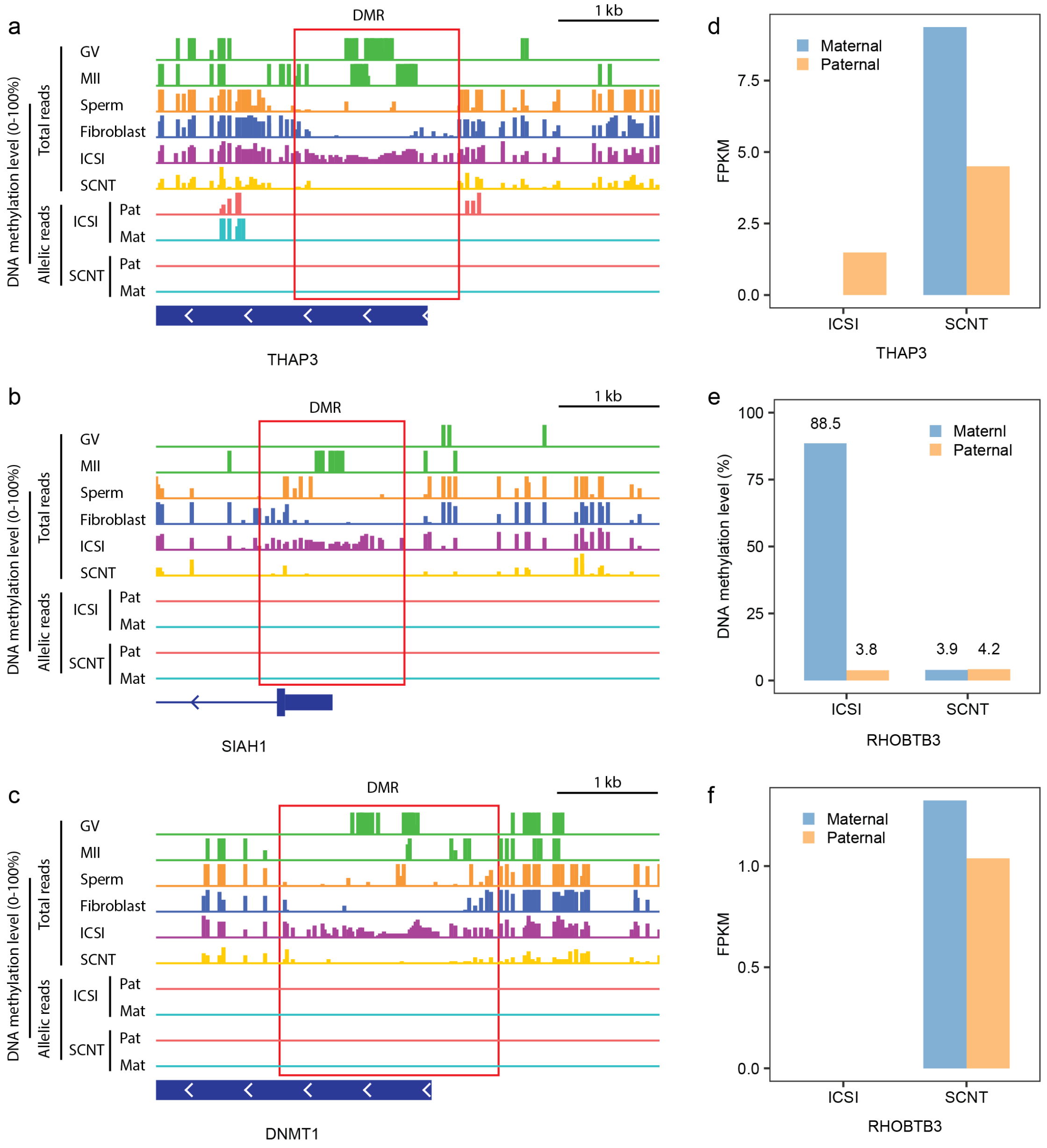

Supplementary Figure 4. **The DNA methylation levels and gene expression levels of the identified imprinted genes.**

a-c Genome browser view showing DNA methylation levels flanking the *THAP3*, *SIAH1* and *DNMT1*. The red box represents the identified DMRs. Mat represents the maternal allele, and Pat represents the paternal allele. Scale bars, 1 kb.

d Bar plot showing the gene expression level (FPKM) of *THAP3* in the parental genomes of ICSI and SCNT blastocysts.

e Bar plot showing the DNA methylation level of *RHOBTB3* in the parental genomes of ICSI and SCNT blastocysts.

f Bar plot showing the gene expression level (FPKM) of *RHOBTB3* in the parental genomes of ICSI and SCNT blastocysts.

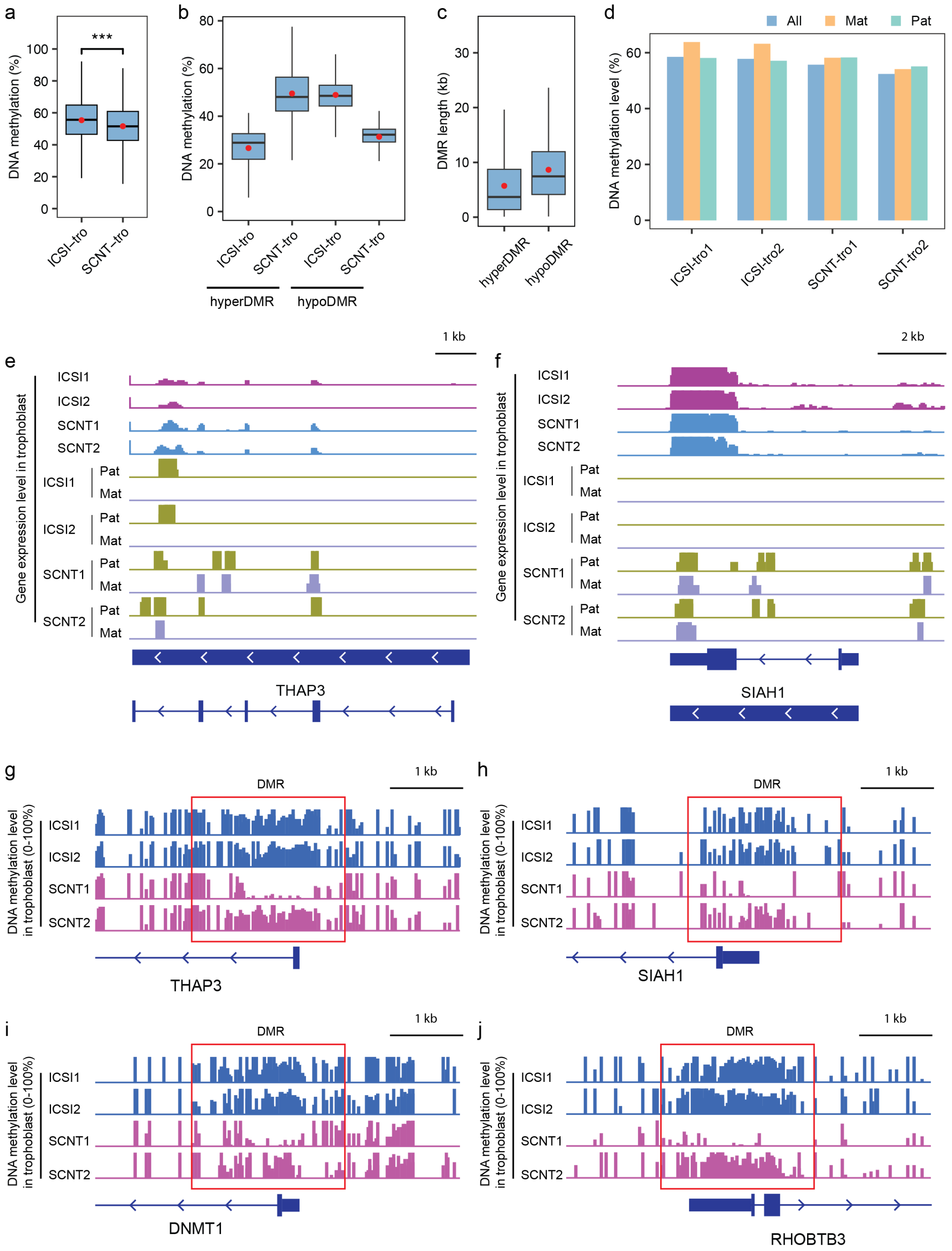

43

44 Supplementary Figure 5. **Identification of ectopically expressed imprinting genes in**

45 **the trophoblast cells of SCNT blastocysts.**

a Boxplot comparing the DNA methylation levels between ICSI and SCNT trophoblast. The average DNA methylation levels were measured in 10 kb windows. The upper and lower edges of the box indicated the 75% and 25% quantiles, while the thick lines in the middle represents the medians of each sample. The red dots indicate the means for each sample. Statistical analysis was performed using the Wilcoxon rank-sum test. *p*-value < 0.001 (***). ICSI-tro, trophoblast cells of monkey ICSI embryos. SCNT-tro, trophoblast cells of monkey SCNT embryos.

b Boxplot showing the DNA methylation levels of hyperDMRs (2,478) and hypoDMRs (6,816) of ICSI and SCNT trophoblasts. The upper and lower edges of the box indicated the 75% and 25% quantiles, while the thick lines in boxes shows the medians. The red dots signify the means for each sample. ICSI-tro, trophoblast cells of monkey ICSI embryos. SCNT-tro, trophoblast cells of monkey SCNT embryos.

c Boxplots comparing the lengths of hyperDMRs and hypoDMRs of ICSI and SCNT trophoblasts. The upper and lower edges of the box indicated the 75% and 25% quantiles, while the thick lines in the middle means the medians of hyper- and hypoDMRs. The red dots stand for the means for each type of sample. ICSI-tro, trophoblast cells of monkey ICSI embryos. SCNT-tro, trophoblast cells of monkey SCNT embryos.

d Bar plot comparing the DNA methylation levels of the whole genome (All), paternal (Pat) and maternal (Mat) genomes of the ICSI and SCNT trophoblast cells.

e Genome browser view illustrating the parental genome expression levels of *THAP3*. Mat, maternal allele. Pat, paternal allele. Scale bar, 1 kb.

f Genome browser view illustrating the parental genome expression levels of *SIAH1*. Mat, maternal allele. Pat, paternal allele. Scale bar, 2 kb.

g-j Genome browser view illustrating the DNA methylation level of the DMRs of *THAP3*, *SIAH1*, *DNMT1* and *RHOBTB3* in the parental genomes of ICSI and SCNT trophoblasts. Scale bars, 1kb.

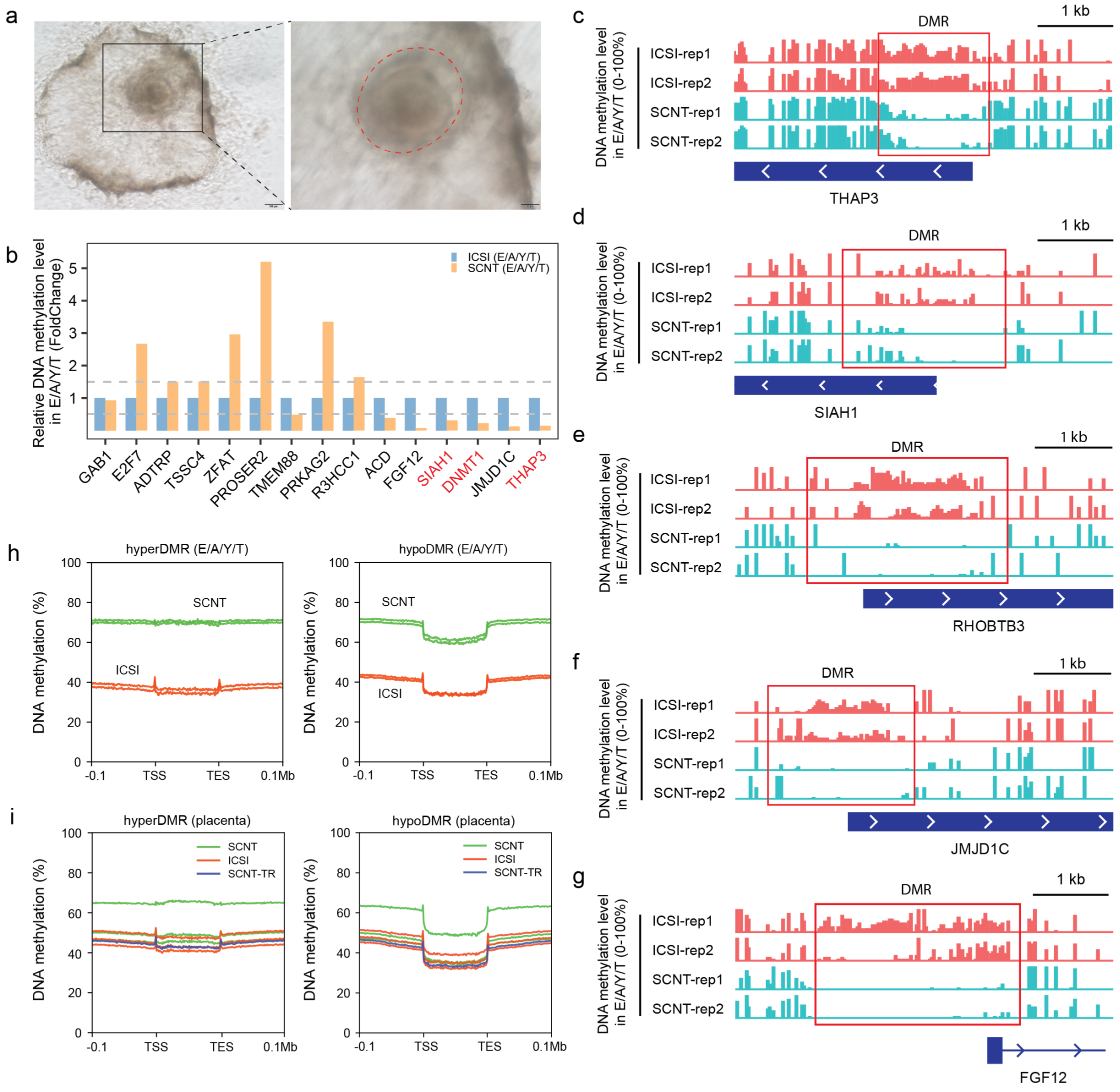

74

75 Supplementary Figure 6. **Continuously imprinting loss in post-implanted monkey**

76 **SCNT embryos.**

a Representative images of *in vitro* cultured post-implanted monkey ICSI embryos on Day 17 post fertilization. The scale bar is 100 μm for left panel and 50 μm for the right panel. The right panel provides a closer observation of the boxed region in the left panel with higher resolution. The E/A/Y/T tissues were outlined with red dotted line in the right panel.

b Bar plot showing the DNA methylation of the DMRs of the imprinting genes (Figure 4c) in E/A/Y/T.

c-g Genome browser view demonstrating the loss of DNA methylation in *THAP3*, *SIAH1*, *RHOBTB3*, *JMJD1C* and *FGF12*. Scale bars, 1 kb.

h Average DNA methylation levels of ICSI and SCNT E/A/Y/T tissues in DMRs compared to their flanking ranges. TSS refers to transcription start sites, and TES refers to transcription end sites.

i Average DNA methylation levels of ICSI, SCNT and SCNT-TR placentas in DMRs compared with their flanking ranges. SCNT-TR in the panel indicates the placenta used for trophoblast replacement. TSS, transcription start sites. TES, transcription end sites.

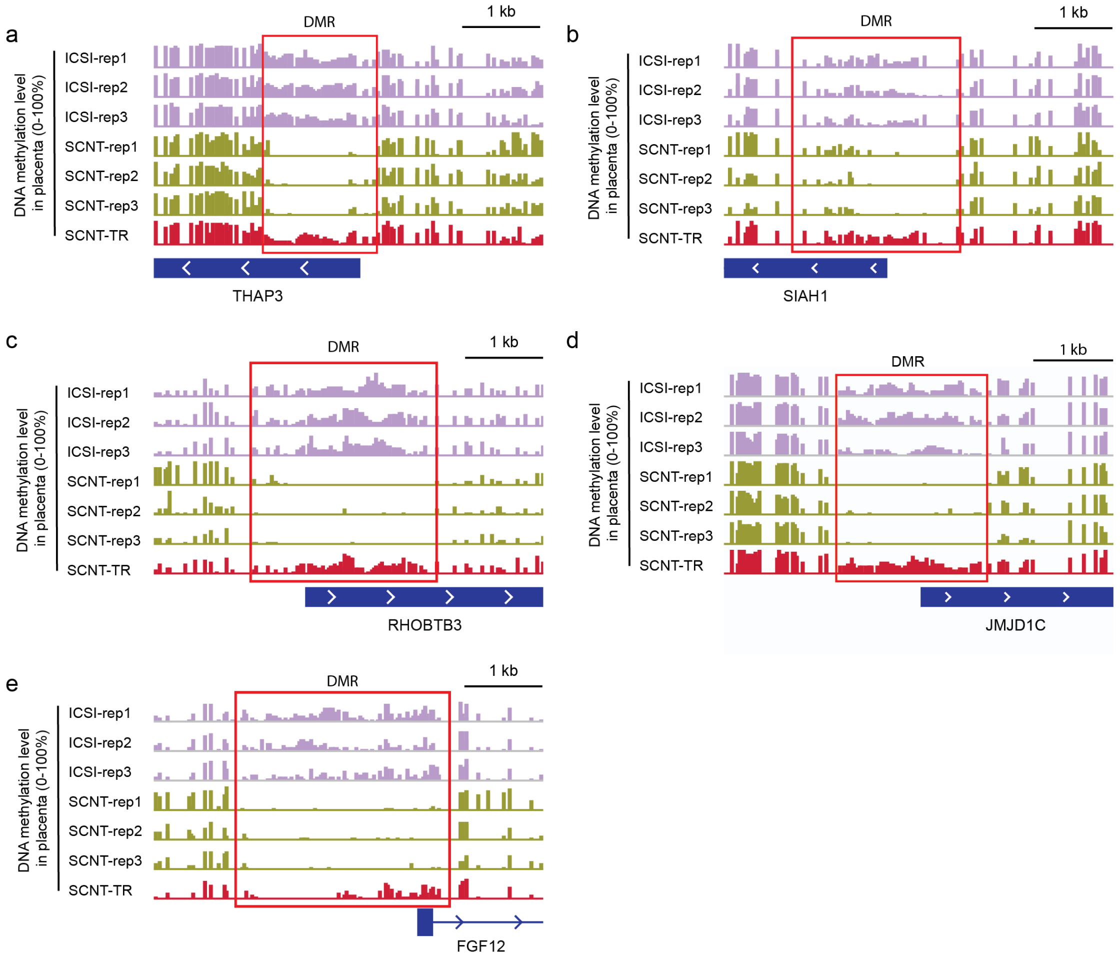

Supplementary Figure 7. **Continuously imprinting loss in monkey SCNT placenta.** **a-e** Genome browser view showing the continuous loss of DNA methylation of *THAP3*, *SIAH1*, *RHOBTB3*, *JMJD1C* and *FGF12* in SCNT placenta and their rescue in the SCNT-TR placenta. Scale bars, 1 kb.

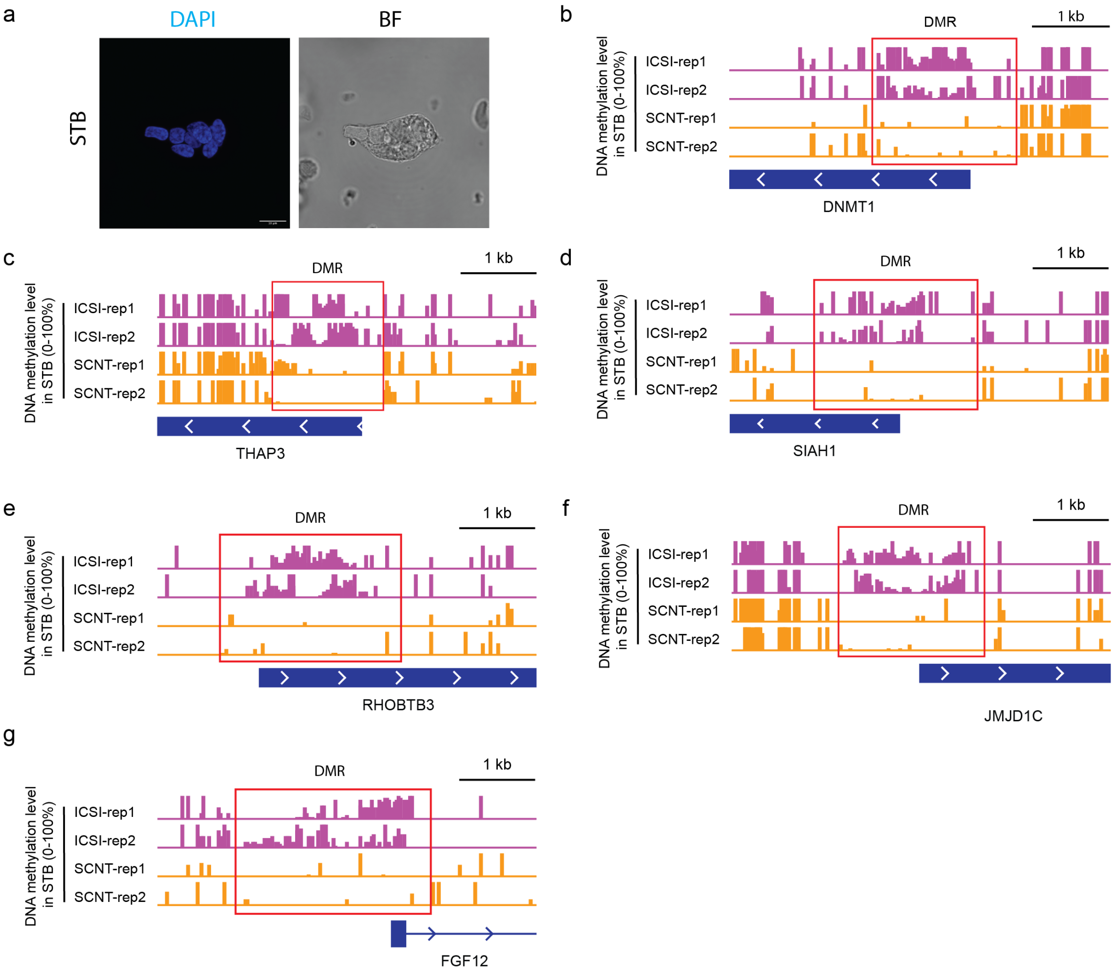

Supplementary Figure 8. **The loss of DNA methylation of candidate imprinting genes in STB cells.**

a DAPI staining showing the nuclei within a single STB cell. BF, bright field. Scale bar, 10 µm.

b-g Genome browser view showing the loss of DNA methylation of *DNMT1*, *THAP3*, *SIAH1*, *RHOBTB3*, *JMJD1C* and *FGF12* in STB cells. Scale bars, 1 kb.

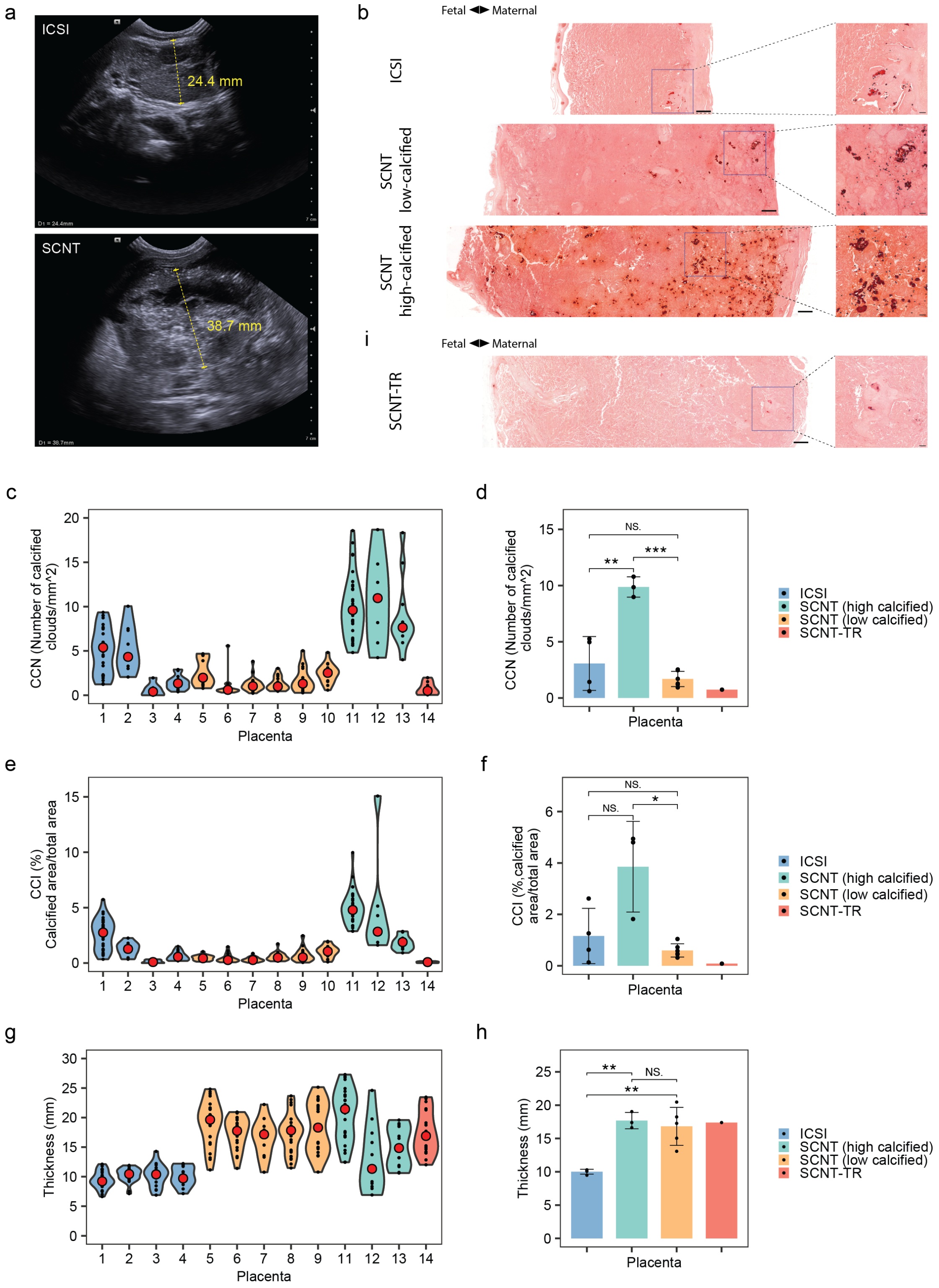

Supplementary Figure 9. **Morphological examination of the monkey placentas.** **a** Ultrasound images of cynomolgus monkey placentas following ICSI and SCNT techniques on gestation day 145 (up) and 130 (down), respectively. Yellow dotted lines indicate the thickness of the placentas.

b Transverse serial sections of ICSI and SCNT placentas (*Macaca fascicularis*) stained with Alizarin red. The high-resolution images on the right are from the blue boxes (3.1 mm x 3.1 mm) in the overview images on the left. The calcified areas are stained deep red as indicated in the slides. The scale bars for overview images are 1 mm, while the scale bars for high-magnification images are 200 μm. The fetal-maternal direction is indicated. The image background was adjusted using ImageJ with the Light Background command.

c Violin plot showing the number of calcification clouds (CCN) in each placenta. The placentas are labeled as numbers 1-14 on the x-axis. Each black dot in the bar indicates a single slide. The red dot in each bar represents the mean CCN of each placenta.

d Bar plot showing the average CCN for ICSI (n=4), lowly calcified SCNT (n=6), highly calcified SCNT (n=3), and SCNT-TR (n=1) placentas. Results are shown as mean ± SD. Each dot in the graph represents a single placenta. Statistical analysis was done using the Student's t-test. The p-values for the non-significant differences (NS.) were > 0.5. Double stars (**) represents the p-value < 0.01. Triple stars (***) represents the p-value < 0.001.

e Violin plot showing the calcification index (CCI) of each placenta. The placentas are labeled as numbers 1-14 on the x-axis. Each black dot in the bar indicates a single slide. The red dots in each bar represents the mean CCN of each placenta.

f Bar plot representing the average CCI for ICSI-full term (n=4), SCNT-full term (n=6), SCNT-abortion (n=3), and SCNT-TR (n=1) placentas. Results are displayed as mean ± SD. Each dot in the graph represents a single placenta. The statistical analyses were done using the Wilcoxon rank-sum test. The p-values for the non-significant differences (NS.) were > 0.5. A single star (*) represents the p-value < 0.05.

g Violin plot illustrating the thickness of each placenta. The placentas are numbered 1-14 on the x-axis. Each black dot within the bar indicates a single slide. The red dots in each bar represents the mean CCN of each placenta.

h Bar plot showing the average thickness of ICSI-full term (n=4), SCNT-full term (n=5), SCNT-abortion (n=3), and SCNT-TR (n=1) placentas determined using transverse serial section slides. Results are shown as mean ± SD. Each dot in the graph represents a single placenta. The statistical analyses were done using the Student's t-test. The p-values for the non-significant differences (NS.) were > 0.5. Double stars (**) represents the p-value < 0.01.

i Transverse serial section of the cloned SCNT-TR placenta of a rhesus monkey. The high-resolution image (right) is from the blue box (3.1 mm x 3.1 mm) in the overview image (left). The calcified areas are highlighted as deep red, as showen in the slides. Scale bar for overview image, 1 mm. Scale bar for high-magnification image, 200 μm. The fetal-maternal direction was indicated as the denotation. The image background was adjusted using ImageJ with the Light Background command.

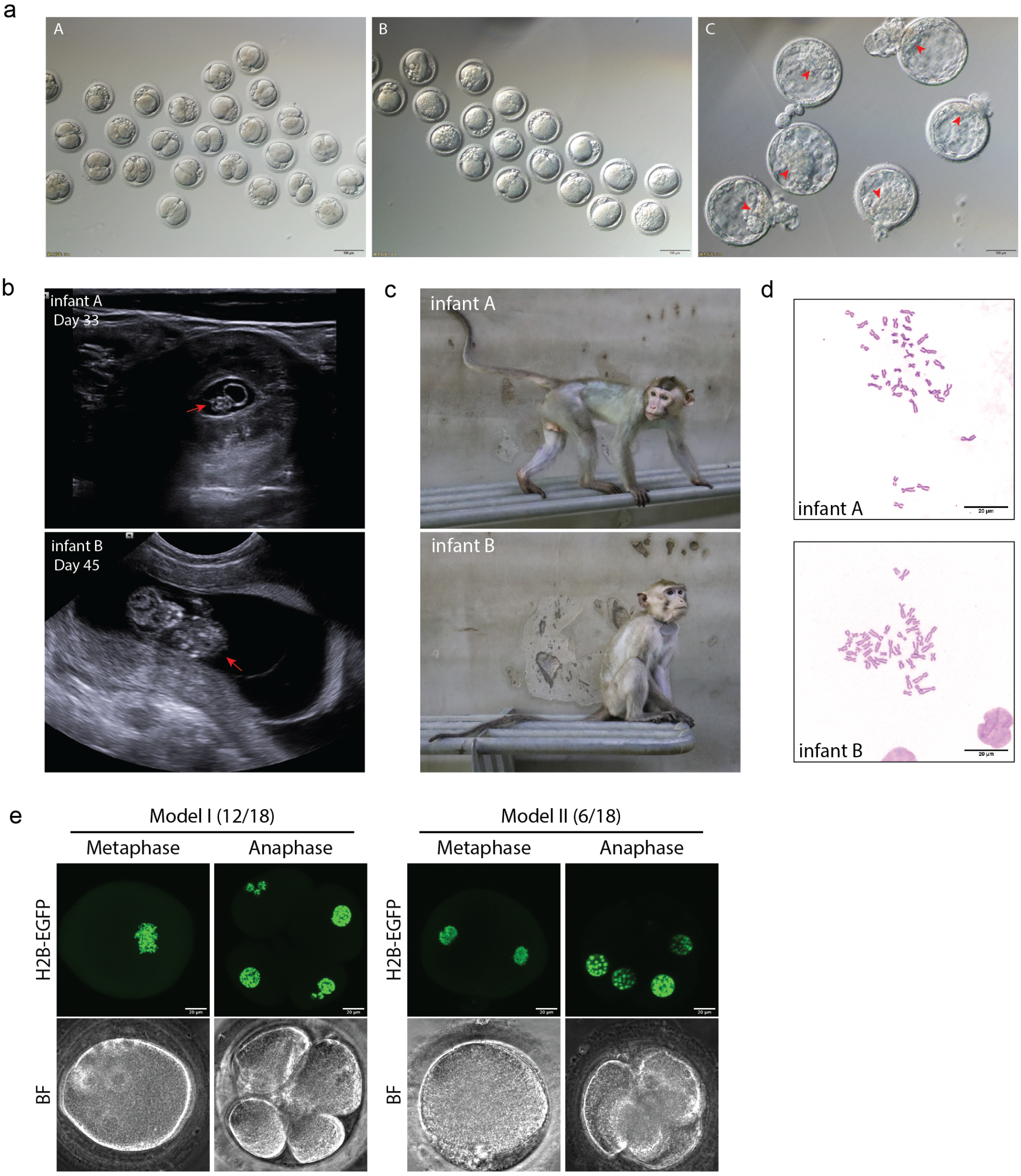

Supplementary Figure 10. **Pre- and post-implant development potential of 2-cell stage electrofused monkey ICSI embryos.**

a A, ICSI embryos at the 2-cell stage; B, Electrofused 2-cell stage ICSI embryos, each embryo containing a single fused blastomere; C, 2-cell-stage-electrofused ICSI embryos at the blastocyst stage. Scale bars, 100 μm.

b Ultrasound examination of the post-implant development of 2-cell-stage-electrofused embryos on day 33 (infant A) and 45 (infant B) of the gestation periods.

c Images of infant A and infant B.

d Karyotype analysis of infant A and infant B. Scale bar, 20 μm.

e Live imaging of the first mitosis of the 2-cell stage fused monkey embryos. Model I, n=12; Model II, n=6. Scale bar, 20 μm.

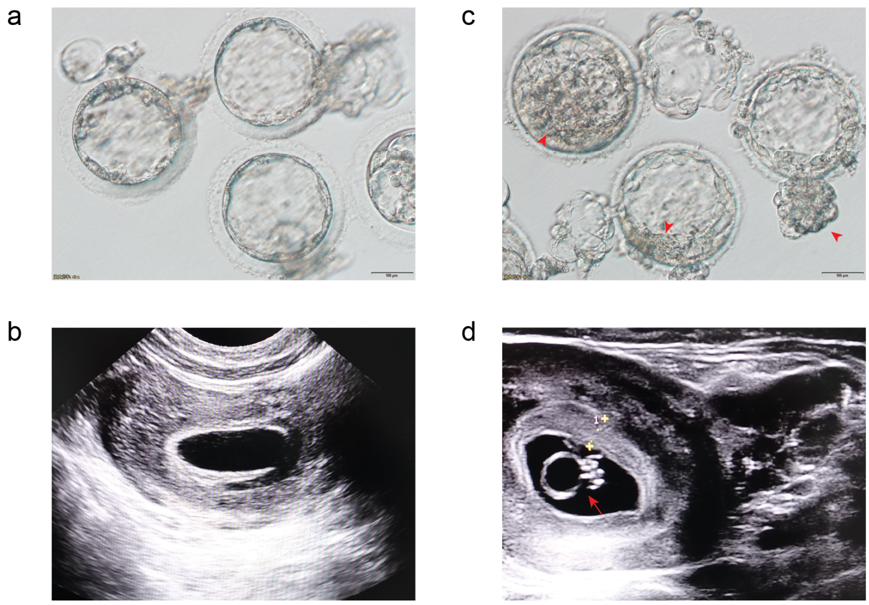

Supplementary Figure 11. **Post-implant development of the ICM-removed ICSI blastocoeles.**

a ICSI blastocoeles without ICMs. Scale bar, 100 µm.

b Ultrasonography of a fetus-free gestational sac (day 28) derived from ICM-removed ICSI blastocoeles.

c ICSI blastocysts with ICMs (red arrowheads). Scale bar, 100 µm.

d Ultrasonography of a gestational sac carrying a fetus (day 28, red arrow).

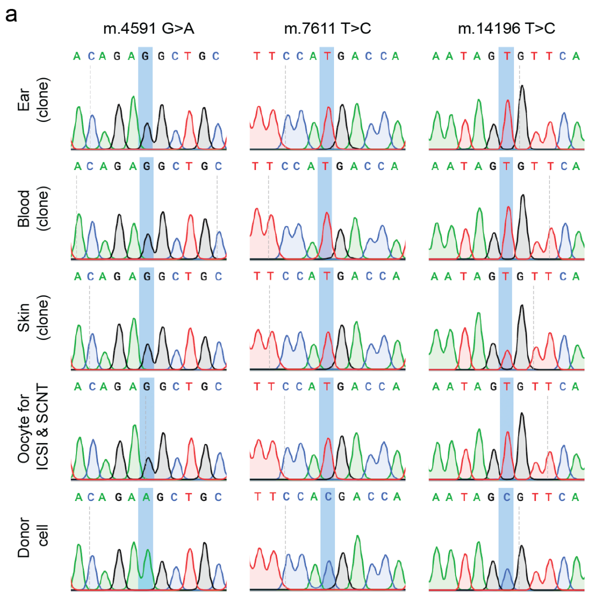

Supplementary Figure 12. **Mitochondrial origin of the cloned rhesus monkey.**

a Representative SNP alleles showing the mtDNA origin of the cloned rhesus monkey.

Supplementary Table 1. Summarize of the samples used for WGBS and RNA-seq.

Sample Type	Datasets	Sample Name	Maternal (♀)[1]	Paternal (♂)[2]	Hybrid type (♀×♂)	Number of embryos for each replication
Blastocyst	WGBS	ICSI-blastocyst-WGBS-rep1	634#	1007207#	cy[3] × rh[4]	11
		ICSI-blastocyst-WGBS-rep2	634#	1007207#	cy × rh	10
		SCNT-blastocyst-WGBS-rep1	18#	1007207#	cy × rh	11
		SCNT-blastocyst-WGBS-rep2	18#	1007207#	cy × rh	12
	RNA-seq	ICSI-blastocyst-RNAseq-rep1	597#	1007207#	cy × rh	2
		ICSI-blastocyst-RNAseq-rep2	683#	1007207#	cy × rh	3
		SCNT-blastocyst-RNAseq-rep1	18#	1007207#	cy × rh	3
		SCNT-blastocyst-RNAseq-rep2	18#	1007207#	cy × rh	1
Trophoblast	WGBS	ICSI-Tro-WGBS-rep1	1010#	1005163#	cy[3] × rh[4]	6~8
		ICSI-Tro-WGBS-rep2	1010#	1005163#	cy × rh	6~8
		SCNT-Tro-WGBS-rep1	5115#	10#	rh × cy	6~8
		SCNT-Tro-WGBS-rep2	5115#	10#	rh × cy	6~8
	RNA-seq	ICSI-Tro-RNAseq-rep1	1010#	1005163#	cy × rh	2~4
		ICSI-Tro-RNAseq-rep2	1010#	1005163#	cy × rh	2~4
		SCNT-Tro-RNAseq-rep1	5115#	10#	rh × cy	2~4
		SCNT-Tro-RNAseq-rep2	5115#	10#	rh × cy	2~4

[1]The number in this column indicates the name of the oocyte donor monkey;
[2]The number in this column indicates the name of the sperm donor monkey;
[3]"cy" is the short for cynomolgus monkey (*Macaca fascicularis*);
[4]"rh" is the short for rhesus monkey (*Macaca mulatta*).

Supplementary Table 2. The identified aberrant imprinted genes with their corresponding DMRs in monkey SCNT blastocysts.

GeneName	Chr	Start	End
ACD	chr20	55834531	55836301
DNMT1	chr19	10452797	10454829
FGF12	chr2	5133102	5135911
JMJD1C	chr9	72565569	72567065
JMJD1C	chr9	72564615	72565234
PRKAG2	chr3	184472967	184479841
PRKAG2	chr3	184548966	184552073
PRKAG2	chr3	184469622	184471698
PRKAG2	chr3	184498437	184499542
PROSER2	chr9	12303906	12306066
PROSER2	chr9	12258138	12261817
PROSER2	chr9	12326560	12330373
R3HCC1	chr8	23651318	23653476
SIAH1	chr20	36038172	36040179
THAP3	chr1	221723668	221725102
TMEM88	chr16	7961392	7962280
TMEM88	chr16	7957989	7958814
TSSC4	chr14	2365111	2367213
ZFAT	chr8	136086665	136090087
ADTRP	chr4	159031354	159047087
ADTRP	chr4	158947201	158956999
ADTRP	chr4	159016574	159021884
E2F7	chr11	76469652	76476469
GAB1	chr5	142676985	142679038

Supplementary Table 3. Summary of the placenta used for morphological examination in this research.

Sample type	Sample Name	CCI (calcification index, %)	Average CCI (%)	CCN (No. of calcified clouds/mm^2)	Average CCN	Thickness (mm)	Average thickness (mm)
ICSI	1	2.62	1.16	4.97	3.06	9.48	10.01
	2	1.26		5.26		10.30	
	3	0.13		0.60		10.21	
	4	0.62		1.42		10.06	
SCNT (low calcified)	5	0.47	0.59	2.53	1.68	19.03	17.71
	6	0.45		0.93		17.39	
	7	0.32		1.20		16.62	
	8	0.58		1.27		17.27	
	9	0.72		1.70		18.24	
	10	1.04		2.47		/*	
SCNT (high calcified)	11	4.94	3.85	9.85	9.87	20.47	16.20
	12	4.80		10.79		13.08	
	13	1.82		8.98		15.04	
SCNT-TR	14	0.08	0.08	0.74	0.74	17.39	17.39

* The data was not available due to the improper sample preparation.

Supplementary Table 4. Post-implant development of hypothetical monkey tetraploid embryos.

Electrofused embryos transferred	Surrogates	Pregnant	Implantation (%)*	Fetus (%)*	Live birth (%)*	Survival (%)*
49	16	8	16 (32.7)	8 (16.3)	3 (6.1)	2 (4.1)

* Data were calculated based on embryos transferred.

Supplementary Table 5. Post-implant development of ICM-removed monkey ICSI blastocoeles.

Blastocoele cavities transferred	Surrogate	Pregnant	Implantation (%)*	Fetus (%)*
83	33	10	10 (12.0)	0 (0.0)

* Data were calculated based on the number of blastocoele cavities transferred.

Supplementary Table 6. Trials of the reconstruction of rhesus SCNT-TR embryos in this study.

Trials	Total embryos activated	Blastocyst used	Blastocyst rate (%)	Reconstructed embryos (ICMs)
1	46	8	17.4	4 (4)
2	16	9	56.3	2 (2)*
3	21	10	47.6	3 (6)
4	17	2	11.8	1 (2)
5	13	3	23.1	1 (3)
Total	113	32	28.3	11 (17)

* The cloned rhesus monkey was generated by the 2nd trial.

Supplementary Table 7. Short tandem repeat analysis of the cloned rhesus monkey derived by trophoblast replacement.

	Donor cell	Rhesus clone	Surrogate	Oocyte for ICSI & SCNT	Sperm donor	Cloned placenta
**D1S548**	192/200	192/200	195/200	195/195	200/200	192/195/200
**D2S149**	204/210	204/210	204/204	204/204	200/210	204/210
**D2S1333**	278/289	278/289	274/297	274/278	289/289	274/278/289
**D3S1768**	184/188	184/188	184/192	184/188	184/188	184/188
**D4S413**	126/140	126/140	128/130	136/138	128/140	126/138/140
**D4S2365**	277/281	277/281	281/281	277/281	277/285	277/281/285
**D5S1457**	124/128	124/128	120/128	124/128	124/124	124/128
**D6S276**	116/124	116/124	114/128	114/126	116/126	114/116/124/126
**D6S291**	202/214	202/214	202/212	198/204	198/214	198/202/214
**D6S501**	175/183	175/183	167/187	175/175	183/187	175/183
**D6S2741**	268/269	268/269	256/273	253/271	268/277	268/269/271/277
**D6S2883**	245/271	245/271	253/268	253/256	236/245	236/245/256/271
**D7S513**	184/194	184/194	177/192	184/198	184/184	184/194/198
**D7S794**	118/135	118/135	106/126	126/135	118/126	118/126/135
**D8S1106**	131/135	131/135	135/135	131/148	131/139	131/135/139
**D9S921**	158/166	158/166	182/190	166/178	158/174	158/166
**D10S1412**	154/157	154/157	148/154	160/166	154/160	154/157/166
**D11S925**	254/279	254/279	277/279	263/263	254/277	254/263/277/279
**D11S2002**	261/261	261/261	253/261	257/261	261/265	257/261
**D12S364**	100/107	100/107	107/120	112/114	100/124	100/107/114/124
**D13S765**	207/215	207/215	175/227	152/207	207/207	152/207/215
**D15S823**	333/350	333/350	316/316	307/361	333/380	316/333/350/361/380
**D16S409**	131/131	131/131	131/131	131/131	131/137	131/131
**D17S1300**	234/288	234/288	226/280	251/257	234/273	234/251/273/288
**D18S537**	158/170	158/170	162/170	162/170	170/170	158/162/170
**D22S685**	309/329	309/329	282/329	309/341	309/357	309/329/341/357
**DXS2506**	276/276	276/276	276/276	265/284	265/265	265/276
**MFGT21**	105/116	105/116	105/121	112/114	107/116	105/107/114/116
**MFGT22**	109/122	109/122	130/132	107/124	109/122	107/109

Supplementary Table 8. Primers used in this study.

Primers for *Kdm4d in vitro* transcription	Forward	TTAATACGACTCACTATAGGGATGGAAACTATGAAGTCTAAGGCCAACT
	Reverse	ATATAAAGACAGCCCGTGGACTTAGG
Primers for H2B-EGFP *in vitro* transcription	Forward	AGCAATGCTCGTTTAGGGAACC
	Reverse	GCGCGCAATTAACCCTCACT
Primers for mtDNA SNPs identification	278F	CATGCAGTTGTTGATCGCACCTA
	278R	GGTTTGGCAAGAGTGGGTT
	4061F	ACAACCACAATCTTCCTAGGCACA
	4061R	GGGGAATGCTGGAGATTGCG
	4591F	CCTGAGAATCCAAACTTCTCCGTG
	4591R	AGCATCCTGATAGTAGGTTGTTGG
	5803F	CTGCAAACACCTACTCTGCATCAA
	5803R	GGCTCAGGGCAGTGCCTATGA
	6040F	TCATAGGCACTGCCCTGAGCC
	6040R	GCGGCTAGGACTGGTAGAGAGAG
	6988F	ATAATCATTGCAATCCCCACCGGT
	6988R	GATGGTGAAGGATGGGTCGTTG
	8680F	ACCCCTTTCTCAACCCCAACAATC
	8680R	TGGTAGGAGGTGGGCTAGGGAG
	9543F	CATAGTTAAACCCAGTCCCTGGCC
	9543R	TCGGAGATGGTGAAGGGTGCTTC
	10051F	ACCACAGGCTTCCACGGAC
	10051R	GGGAGGTTTGTTGTTTGAATGGCT
	11530F	GAGACCACTCTCATTCCTACCCT
	11530R	TGTTTTCTCGTGTGAAGGGGGG
	13628F	CCCTTCCTCACAGGCTTCTACTCC
	13628R	GTTAGTGGTGTGGTTGGTTGTGTG
	15129F	GGACTCCAACCATAACTAACGGCA
	15129R	GCTGTCAATGGCGTATCCTCCTC
	16038F	CACTATCGGCCAAGTAGCATCCAT
	16038R	GCATCCGTGGTGAGGAGGATTAT
Primers for X chromosome SNPs identification	X9-F	TGGTTTCCTGAGATTACCGCAA
	X9-R	TTATGGTGCCCCCATCAGTTAC
	X15-F	GGTCCATCTGTGTCCTTGAACT
	X15-R	ATTTGCAGCGAGGAAAGACAAC
Deep sequencing primers for X chromosome SNPs of rhesus clone	deepseq-X9-F	ggagtgagtacggtgtgcGTCACCCTTGCCATTTTGTTCC
	deepseq-X9-R	gagttggatgctggatggAGTTTAATGCTGACTCTGCTGG
	deepseq-X15-F	ggagtgagtacggtgtgcCATGGAATTCAATACACCCCAATG
	deepseq-X15-R	gagttggatgctggatggATTTGCAGCGAGGAAAGACAAC

Supplementary Table 9. Software and packages used in this study for RNA-seq, WGBS and WGS data processing.

ABI PRISM 3730 genetic analyzer	ABI
ImageJ	https://imagej.nih.gov/ij/
fastp (version 0.23.2)	https://github.com/OpenGene/fastp
Bismark (version 0.23.1)	https://www.bioinformatics.babraham.ac.uk/projects/bismark/
Hisat2 (version 2.2.1)	https://github.com/DaehwanKimLab/hisat2
SAMtools (version 1.15.1)	https://github.com/samtools/samtools
featureCounts (version 2.0.1)	https://subread.sourceforge.net/featureCounts.html
Deeptools (version 3.5.1)	https://deeptools.readthedocs.io/en/develop/
Bowtie2 (version 2.4.5)	https://bowtie-bio.sourceforge.net/bowtie2/index.shtml
SNPsplit (version 0.5.0)	https://www.bioinformatics.babraham.ac.uk/projects/SNPsplit/
methpipe (version 3.4.3)	https://github.com/smithlabcode/methpipe
methylKit	https://github.com/al2na/methylKit
HOMER (version 4.11)	http://homer.ucsd.edu/homer/
edgeR	https://bioinf.wehi.edu.au/edgeR/
BWA (version 0.7.17-r1188)	https://hpc.nih.gov/apps/bwa.html
GATK (version 4.2.6.1)	https://github.com/broadinstitute/gatk
sentieon (Release 202112.05)	https://www.sentieon.com
BEDtools (version 2.30.0)	https://bedtools.readthedocs.io/en/latest/index.html
R (version 4.2.1)	https://www.r-project.org
Trim_galore (version 0.6.7)	https://www.bioinformatics.babraham.ac.uk/projects/trim_galore/
