# Supplementary material for: Reprogramming mechanism dissection and trophoblast replacement application in monkey somatic cell nuclear transfer
Source: Nat Commun. 2024 Jan 16;15:5. doi: 10.1038/s41467-023-43985-7 (PMC10791636; doi:10.1038/s41467-023-43985-7)
Supplement: Supplementary file 3 — Description of Additional Supplementary Files [file 41467_2023_43985_MOESM3_ESM.pdf]

<u>Description of Additional Supplementary Files</u>

Supplementary Movie 1. The trophoblast replacement process among monkey blastocysts.
