# Supplementary material for: Reprogramming mechanism dissection and trophoblast replacement application in monkey somatic cell nuclear transfer
Source: Nat Commun. 2024 Jan 16;15:5. doi: 10.1038/s41467-023-43985-7 (PMC10791636; doi:10.1038/s41467-023-43985-7)
Supplement: Supplementary file 5 — Reporting Summary [file 41467_2023_43985_MOESM5_ESM.pdf]

nature portfolio

Corresponding author(s): Qiang Sun
Zhen Liu
Falong Lu

Last updated by author(s): Oct 8, 2023

Reporting Summary

Nature Portfolio wishes to improve the reproducibility of the work that we publish. This form provides structure for consistency and transparency in reporting. For further information on Nature Portfolio policies, see our Editorial Policies and the Editorial Policy Checklist.

Statistics

For all statistical analyses, confirm that the following items are present in the figure legend, table legend, main text, or Methods section.

n/a	Confirmed	
☐	☒	The exact sample size (n) for each experimental group/condition, given as a discrete number and unit of measurement
☐	☒	A statement on whether measurements were taken from distinct samples or whether the same sample was measured repeatedly
☐	☒	The statistical test(s) used AND whether they are one- or two-sided<br>*Only common tests should be described solely by name; describe more complex techniques in the Methods section.*
☐	☒	A description of all covariates tested
☐	☒	A description of any assumptions or corrections, such as tests of normality and adjustment for multiple comparisons
☐	☒	A full description of the statistical parameters including central tendency (e.g. means) or other basic estimates (e.g. regression coefficient) AND variation (e.g. standard deviation) or associated estimates of uncertainty (e.g. confidence intervals)
☐	☒	For null hypothesis testing, the test statistic (e.g. F, t, r) with confidence intervals, effect sizes, degrees of freedom and P value noted<br>*Give P values as exact values whenever suitable.*
☒	☐	For Bayesian analysis, information on the choice of priors and Markov chain Monte Carlo settings
☒	☐	For hierarchical and complex designs, identification of the appropriate level for tests and full reporting of outcomes
☐	☒	Estimates of effect sizes (e.g. Cohen's d, Pearson's r), indicating how they were calculated

Our web collection on statistics for biologists contains articles on many of the points above.

Software and code

Policy information about availability of computer code

Data collection	The images of histological examination of placentas using alizarin red staining were acquired using Olympus VS120.<br>Images of the placental histological examination were processed using Fiji (version 2.3.0/1.53q).<br>The sequencing data of blastocyst RNA-seq, WGBS and CUT&RUN were generated using Illumina NovaSeq 6000 sequencer.<br>The WGBS data of oocyte and placenta were generated using DNBseq platform.
Data analysis	The sequencing data were processed using softwares including fastp (version 0.23.2), Bismark (version 0.23.1), Hisat2 (version 2.2.1), SAMtools (version 1.15.1), featureCounts (version 2.0.1), deepTools (version 3.5.1), Bowtie2 (version 2.4.5), SNPsplit (version 0.5.0), methpipe (version 3.4.3), BWA (version 0.7.17-r1188), BEDtools (version 2.30.0), sentieon (Release 202112.05), GATK (version 4.2.6.1), HOMER (version 4.11) with default parameters.<br>The packages of edgeR and methylKit were used for the down stream analyses of RNA-seq and WGBS datasets.<br>The site-specific deep sequencing results were obtained using the Hi-TOM platform.<br>The Sanger sequencing data were analyzed using SnapGene (version 4.3.6).<br>All of the statistic analyses were performed using R (version 4.2.1).

For manuscripts utilizing custom algorithms or software that are central to the research but not yet described in published literature, software must be made available to editors and reviewers. We strongly encourage code deposition in a community repository (e.g. GitHub). See the Nature Portfolio guidelines for submitting code & software for further information.

Data

Policy information about availability of data

All manuscripts must include a data availability statement. This statement should provide the following information, where applicable:

- Accession codes, unique identifiers, or web links for publicly available datasets
- A description of any restrictions on data availability
- For clinical datasets or third party data, please ensure that the statement adheres to our policy

The RNA-seq datasets of hybrid blastocyst and trophoblasts generated in this study have been deposited in the Gene Expression Omnibus (GEO) database under accession code GSE221634 [https://www.ncbi.nlm.nih.gov/geo/query/acc.cgi?acc=GSE221634] and GSE239741 [https://www.ncbi.nlm.nih.gov/geo/query/acc.cgi?acc=GSE239741]. The WGBS datasets of hybrid blastocyst and trophoblasts generated in this study have been deposited in the GEO database under accession code GSE221636 [https://www.ncbi.nlm.nih.gov/geo/query/acc.cgi?acc=GSE222636] and GSE239742 [https://www.ncbi.nlm.nih.gov/geo/query/acc.cgi?acc=GSE239742]. The WGBS dataset of hybrid fibroblast generated in this study has been deposited in the GEO database under accession code GSE221637 [https://www.ncbi.nlm.nih.gov/geo/query/acc.cgi?acc=GSE221637]. The WGBS datasets for placenta, oocyte, STB cells and in vitro cultured post-implanted monkey embryos generated in this study have been deposited in the GEO database under the accession code GSE222930 [https://www.ncbi.nlm.nih.gov/geo/query/acc.cgi?acc=GSE222930]. The whole genome sequencing datasets of parental genomes generated in this study have been directly deposited in the Sequence Read Archive (SRA) under the BioProject number of PRJNA915580 [https://www.ncbi.nlm.nih.gov/bioproject/?term=PRJNA915580]. The public WGBS data of human sperm used in this study is available in the GEO database under accession code GSE109344 [https://www.ncbi.nlm.nih.gov/geo/query/acc.cgi?acc=GSE109344]. The raw data used for statistical graphs in this study is available in the Source Data folder of figshare [https://doi.org/10.6084/m9.figshare.23957904].

Human research participants

Policy information about studies involving human research participants and Sex and Gender in Research.

Reporting on sex and gender	There is no human participants in this research.
Population characteristics	There is no human participants in this research.
Recruitment	There is no human participants in this research.
Ethics oversight	There is no human participants in this research.

Note that full information on the approval of the study protocol must also be provided in the manuscript.

Field-specific reporting

Please select the one below that is the best fit for your research. If you are not sure, read the appropriate sections before making your selection.

☒ Life sciences ☐ Behavioural & social sciences ☐ Ecological, evolutionary & environmental sciences

For a reference copy of the document with all sections, see nature.com/documents/nr-reporting-summary-flat.pdf

Life sciences study design

All studies must disclose on these points even when the disclosure is negative.

Sample size	For the histological examination of placentas, at least 3 placentas were included for the ICSI and SCNT groups. However, there is only one SCNT-TR placenta was included because we only obtained one SCNT-TR placenta in this research.<br>For the RNA-seq, WGBS and CUT&RUN datasets of ICSI and SCNT embryos, 2 replicates were included for each group.<br>For the WGBS of ICSI and SCNT placentas, 3 replicates were included for each group.<br>For the site-specific deep sequencing of the cloned rhesus monkey, 2 SNPs were sequenced using the next-generation sequencing method.
Data exclusions	The 10# placenta in Extended Data Figure 9g and h was excluded because of improper sample preparation. This placenta was cross sectionally cut into two parts, so we cannot have the data of its thickness.
Replication	In order to verify the reproducibility of the the experimental findings, at least 2 replicates were included in each experiments. And all attempts at replication were successful.
Randomization	The blastocysts of ICSI and SCNT were randomly allocated into each replicates when performing sample collecting.<br>Both the ICSI and SCNT placentas were randomly chosen in this research.
Blinding	Blinding was not required in this research.

Reporting for specific materials, systems and methods

We require information from authors about some types of materials, experimental systems and methods used in many studies. Here, indicate whether each material, system or method listed is relevant to your study. If you are not sure if a list item applies to your research, read the appropriate section before selecting a response.

Materials & experimental systems

n/a	Involved in the study
☐	☒ Antibodies
☐	☒ Eukaryotic cell lines
☒	☐ Palaeontology and archaeology
☐	☒ Animals and other organisms
☒	☐ Clinical data
☒	☐ Dual use research of concern

Methods

n/a	Involved in the study
☒	☐ ChIP-seq
☒	☐ Flow cytometry
☒	☐ MRI-based neuroimaging

Antibodies

Antibodies used

anti-∂-tubulin (1:200, abcam, ab80779), anti-pH3 (1:100, abcam, ab5176), Alexa Fluor® 488-conjugated AffiniPure Donkey Anti-Mouse IgG (H+L) (1:5000, JackSon ImmunoResearch, 715-545-150), Alexa Fluor Cy3-conjugated AffiniPure Donkey Anti-Rabbit IgG (1:5000, JackSon ImmunoResearch, 711-165-152) and DAPI (1:5000).

Validation

anti-∂-tubulin:
ab80779
supplier: Abcam
host: mouse
clonality: Monoclonal,
conjugate: unconjugated
reactivity: Homo sapiens (human), Mus musculus (house mouse), Saccharomyces cerevisiae (yeast)
Applications: WB, ICC-IF, SW-Size, EM

anti-pH3:
ab5176
supplier: Abcam
host: rabbit
clonality: polyclonal,
conjugate: unconjugated
reactivity: Homo sapiens (human)
Applications: WB, IHC-P, ICC

Alexa Fluor® 488-conjugated AffiniPure Donkey Anti-Mouse IgG (H+L)
715-545-150
supplier: JackSon ImmunoResearch
Target: Mouse
Host: Donkey
Antibody Format: Whole IgG
Specificity: IgG (H+L)
Minimal Cross Reactivity: Bovine, Chicken, Goat, Guinea Pig, Syrian Hamster, Horse, Human, Rabbit, Sheep Serum Proteins
Conjugate: Alexa Fluor® 488
Product Category: Whole IgG Affinity-Purified Antibodies
Clonality: Polyclonal
RRID: AB_2340846

Alexa Fluor Cy3-conjugated AffiniPure Donkey Anti-Rabbit IgG
711-165-152
supplier: JackSon ImmunoResearch
Target: Rabbit
Host: Donkey
Antibody Format: Whole IgG
Specificity: IgG (H+L)
Minimal Cross Reactivity: Bovine, Chicken, Goat, Guinea Pig, Syrian Hamster, Horse, Human, Mouse, Rat, Sheep Serum Proteins
Conjugate: Cyanine Cy™3
Product Category: Whole IgG Affinity-Purified Antibodies
Clonality: Polyclonal
RRID: AB_2307443

Eukaryotic cell lines

Policy information about cell lines and Sex and Gender in Research

Cell line source(s)	The somatic cell donor cell line (rh180502-1) is a male cell line, which is in house made.
Authentication	The cell line used in this study is not authenticated.
Mycoplasma contamination	The cell line was tested negative for mycoplasma contamination.
Commonly misidentified lines (See ICLAC register)	There is no commonly misidentified cell lines used in this study.

Animals and other research organisms

Policy information about studies involving animals; ARRIVE guidelines recommended for reporting animal research, and Sex and Gender in Research

Laboratory animals	Rhesus monkey, cynomolgus monkeys, and hybrids of rhesus and cynomolgus monkeys were utilized in this study, all falling within their reproductive age.<br><br>To provide more detailed information, for the somatic cloning of rhesus monkeys, the 7 surrogate mothers ranged in age from 9 to 11 years old, the 6 oocyte donor monkeys were aged between 8 and 11 years old, and the 2 sperm donor monkeys were 8 and 10 years old, respectively.<br><br>In the construction of tetraploid embryos and blastocoele cavity transfer experiments, the 49 surrogate mothers ranged in age from 4 years old to 14 years old, the oocyte donors were between 6 and 15 years old, while the two sperm donors were 11 and 16 years old, respectively.<br><br>Two hybrid monkeys, age 1 and 5 years old, served as the somatic cell donors for generating hybrid SCNT embryos.
Wild animals	This study did not involve wild animals.
Reporting on sex	Monkeys of both sexes were utilized in this study. Specifically, female monkeys were used as oocyte donors and surrogate mothers, while male monkeys were employed as sperm donors. Sperm of rhesus monkeys was injected into the oocytes of cynomolgus monkeys to generate hybrid monkey and embryos, and vice versa. The donor cell line for rhesus monkey cloning were obtained from an aborted male fetus. Due to the aforementioned reasons, we do not consider sex a character that influences the outcome of our study; therefore, we did not collect the sex-based data in this study.
Field-collected samples	This study did not involve samples collected from the field.
Ethics oversight	All animal procedures followed the guidelines of the Animal Use and Care Committees in the Shanghai Institute of Biological Science, Chinese Academy of Sciences (CAS), and Institute of Neuroscience, CAS Center for Excellence in Brain Science and Intelligence Technology. The application approved by the committees is entitled “The research of constructing macaque animal model by somatic cell nuclear transfer” (ION-2018002R01).

Note that full information on the approval of the study protocol must also be provided in the manuscript.
